# High Levels of Thromboxane (TX) Are Associated with the Sex-Dependent Non-Dipping Phenomenon in Ischemic Stroke Patients

**DOI:** 10.3390/jcm11092652

**Published:** 2022-05-09

**Authors:** Arleta Drozd, Małgorzata Szczuko, Andrzej Bohatyrewicz, Alina Jurewicz, Dariusz Kotlęga

**Affiliations:** 1 Department of Human Nutrition and Metabolomics, Pomeranian Medical University in Szczecin, 71-460 Szczecin, Poland; arleta.drozd@pum.edu.pl; 2 Department of Orthopaedics Traumatology and Muskuloskeletal Oncology, Pomeranian Medical University in Szczecin, 71-252 Szczecin, Poland; andrzej.bohatyrewicz@pum.edu.pl (A.B.); alina.jurewicz@pum.edu.pl (A.J.); 3 Department of Pharmacology and Toxicology, University of Zielona Góra, 65-001 Zielona Góra, Poland; dkotlega@uz.zgora.pl

**Keywords:** ischemic stroke, dipping, non-dipping, hypertension, thromboxane

## Abstract

Background: Inflammation and high blood pressure (nondipping profile) during the rest/sleep period have been associated with an effect on the incidence of cardiovascular disorders and a more severe course in the ischemic cerebrovascular event. There are no available data on the relationship between dipping status and the pro-inflammatory metabolites of arachidonic acid (AA); therefore, we undertook a study to investigate the influence of thromboxane on the incidence of nondipping among patients after stroke. Methods: Sixty-two patients with ischemic stroke (including 34 women and 28 men) were tested for the involvement of thromboxane in the nondipping phenomenon. Subjects were analyzed for the presence of the physiological phenomenon of dipping (DIP group) versus its absence—nondipping (NDIP group). Thromboxane (TX) measurements were performed using liquid chromatography, and blood pressure was measured 24 h a day in all subjects. Results: The analysis of the thromboxane level in the plasma of patients after ischemic stroke showed significant differences in terms of sex (*p* = 0.0004). Among women in both groups, the concentration of TX was high, while similar levels were observed in the group of men from the NDIP group. However, when comparing men in the DIP and NDIP groups, a lower TX level was noticeable in the DIP group. Conclusions: A higher level of TX may be associated with a disturbance of the physiological phenomenon of DIP in men and women. However, in our opinion, TX is not the main determinant of the DIP phenomenon and, at the same time, other pro-inflammatory factors may also be involved in the occurrence of this singularity.

## 1. Introduction

Cardiovascular diseases (CVDs) are one of the leading causes of morbidity and mortality in many developer countries [1]. They are distinguished by chronic and latent development and a constant potential threat to life [1,2]. Therefore, it is necessary to undertake research to better understand the mechanism. Stroke is considered to be an age-related disease, but there are many modifiable and non-modifiable risk factors. Gender is related to stroke, with young women having an equal or higher risk of stroke than men, while men are at higher risk in old age. Racial factors also play a role in the development of stroke, as African Americans are at twice the risk compared to Caucasians [3]. Moreover, smaller nocturnal blood pressure falls and a higher prevalence of non-dipping may contribute to the higher levels of hypertension complications seen in Black people. No such phenomenon was seen in South Asian subjects [4].

### 1.1. Stroke Terminology and Mechanism of the Cellular Reaction

The term stroke includes subarachnoid hemorrhage (5%), intracerebral hemorrhage (15%), and ischemic stroke, accounting for approximately 80% of cases [5]. The cells that penetrate the hypoxic tissue most quickly are neutrophils [6]. Neutrophils begin the process of penetrating the inflammation focus in the first hours of the infarction and have a significant impact on the enlargement of the area of the cerebral infarction. Monocytes accumulate in the first 24 h, peaking at 48 h. The lymphocytes arrive at the very end, roughly 24–48 h after the stroke begins [7]. In the following days, monocytes and macrophages migrate there. The inflammatory cells damage the nervous tissue through the production of free radicals, cytokines and nitric oxide [8].

The oxidative stress caused by hypoxia and the lipid peroxidation following the ischemic cascade release arachidonic acid (AA) from cell membranes. The highest concentrations of arachidonic acid are found in the brain, muscles, liver, spleen and retina, circulating free AA concentrations in the body are usually very low due to albumin binding.

The endogenous production of AA occurs mainly through the release of AA from cell membrane phospholipids in the re action catalyzed by phospholipase A2 (PLA2). The process is induced by a variety of cell activation signals, including the stimulation of inflammation or infection of the tumor necrosis factor receptor (TNFR) and the stimulation of the Toll-like receptor 4 (TLR4). Free arachidonic acid converts to prostaglandin G/H by cyclooxigenase (COX), and then thromboxane A2 (TXA2) is produced [7]. TXA2 and prostaglandins (PGs) are metabolites of the COX1 pathway and alter vascular tone and mediate platelet aggregation.

The COX pathway is one of the main goals in the treatment of atherosclerosis and ischemic heart disease, because it influences the main pathophysiological features of these diseases, including platelet aggregation, vascular tone and inflammatory processes in atherosclerotic lesions [9]. The anti-inflammatory and anticoagulant features of aspirin, the only known irreversible inhibitor of platelet COX1, are primarily associated with the inhibition of PG and TXA2 synthesis. Therefore, the search for other mechanisms and modulation pathways is justified. The measurement of serum thromboxane B2 (TXB2), a stable metabolic product of TXA 2, is the only test that measures the effect of aspirin on platelet COX-1 activity. The measurement of serum TXB2 in whole blood allowed to clot determines the maximum platelet production of TXA 2 [10,11].

The epidemiological role of TXB2 was presented to be a potential risk factor for ischemic stroke. Moreover, TXA2 synthesis is inhibited by estrogens, which may be of importance in the sex-related differences in inflammation and risk of stroke [12].

### 1.2. The Phenomenon of Non-Dipping and Implications for Cardiovascular Diseases

Recent research on the determinants of ischemic stroke inclines towards the phenomenon known as nondipping. This mechanism is associated with a significant risk of myocardial infarction and ischemic stroke [13,14].

The nondipping phenomenon is when a physiological, nocturnal lowering blood pressure effect is lacking. The physiological dipping effect is under control of the suprachiasmatic nuclei of the anterior hypothalamus. Physiological rhythm and a lack of dipping is associated with increased cardiovascular disorders and more severe end-organ damage [15,16]. This physiological process determines a reduction in both systolic and diastolic pressure during sleep by 10–20% [17]. The state of nondipping also plays an important role in cardiovascular risk in people without hypertension, contributing to an increased risk of cardiovascular mortality and more serious organ damage [18]. Data on the thickness of the intima-media diameter in the common carotid artery (intima-media thickness, IMT) and atherosclerotic plaque in non-dipping and dipping subjects assessed by ultrasound were analyzed. The relationship (in a group of 2753 patients) was confirmed between non-dipping pattern and an increased risk of subclinical lesions in the carotid artery [19]. The meta-analysis supports the view that effective blood pressure control throughout the day, especially at night, can prevent the progression of vascular damage associated with the non-dipping phenotype [19]. Another meta-analysis provided new insight into the unfavorable relationship between reversed dipping pattern and subclinical heart changes. The group with inverted dipping status had an increased risk of left ventricular hypertrophy compared to the preserved physiological phenomenon of dipping. The detection of this blood pressure phenotype may identify individuals at increased risk of subclinical organ damage [20,21].

There are no data available on the relationship between the dipping status and the pro-inflammatory metabolites of arachidonic acid in both the healthy population, and even more so in ischemic stroke patients. There is also very limited information on the role of non-dipping in the pathogenesis of stroke, and it undoubtedly appears to be related. Pro-inflammatory mediators, including thromboxane derived from the arachidonic acid pathway, associated with high cardiovascular risk, in our opinion, may also be related to the nondipping phenomenon. Therefore, we have undertaken research in this direction, taking into account differences in particular in terms of gender, age and other risk factors for cardiovascular diseases.

## 2. Materials and Methods

### 2.1. Study Design and Population

In the study, sixty-two ischemic stroke patients were included (34 women and 28 men). Patients (Caucasians) were hospitalized in the Neurology Department in the district hospital in Głogów, Poland.

The inclusion criteria were the diagnosis of the ischemic stroke established on the basis of clinical symptoms and additional tests results, including brain imaging (computerized tomography or magnetic resonance imaging (MRI) scans), and who received treatment in accordance with standard protocols and guidelines [22]. The exclusion criteria included intracranial hemorrhage visible in brain imaging, symptoms of active infection (temperature over 37.4 °C), clinical or biochemical symptoms of infection, an active autoimmune disorder or malignancy and speech or consciousness impairment due to cerebral or metabolic causes. We obtained informed consent from the patients. During hospitalization, all patients were treated with statins and acetylsalicylic acid, and previous hypotensive treatment was maintained. The group consisted of 33 patients with preserved physiological dipping effect (DIP) and 31 patients with nondipping phenomenon (NDIP). The characteristics of both groups are presented in Table 1.

All patients had 24 h blood pressure measurement (ABPM). ABPM tests were performed between 4 and 6 days after the onset of a stroke. BP reading were recorded at 15 min intervals during the daytime and at 30 min intervals during nighttime. Daytime and nighttime were defined as 6 am to 10 pm and from 10 pm to 6 am, respectively. The recordings were analyzed using dedicated software, and patients were excluded from the study if ≥20% of the measurements were not recorded successfully. Patients with mean BP decline of ≥10% during nighttime were defined as dippers, whereas those with a recorded decline of <10% were considered nondippers [23].

### 2.2. Detection of Fatty Acids Metabolites

The mobile phase was a mixture of double-distilled water (Milli-Q Water System) and methanol (cat. no. 1.06018) with the addition of acetic acid (cat. no. 543808) HPLC grade, from Sigma Aldrich (Tokyo, Japan). The buffers used for HPLC analysis were filtered through 0.22 µm nylon filters (Cat no. 9301-0895, Agilent Technologies, Cheadle, UK). Thromboxane (cat. no. 19030, Cayman Chemical, Michigan, USA) was extracted from the 0.5 mL of plasma by using a solid-phase extraction RP-18 SPE columns (Agilent Technologies, Cheadle, UK).

The HPLC separations were performed on the 1260 liquid chromatograph (Agilent Technologies), degasser model G1379B, bin pump model G1312B, column oven model G1316A and G1315CDAD VL+. Samples were injected using model G1329B. The Agilent ChemStation software (Agilent Technologies, Cheadle, UK) was used for data acquisition, instrument control and analysis. The temperature of column oven was set at 21 °C. The separation was completed on Thermo Scientific Hypersil BDS C18 column 100 × 4.6 mm 2.4 µm (cat no. 28102–154630).

A gradient method was used, with a mixture of solvent A (methanol/water/acetic acid, 50/50/0.1, *v*/*v*/*v*) and B (methanol/water/acetic acid, 100/0/0.1, *v*/*v*/*v*) that constituted the mobile phase. The buffer B percentage in the mobile phase was 30% at 0.0 min to 2.00 min of separation, which increased linearly to 80% at 33 min, 98% (33.1–37.5 min) and 30% (40.3–45 min). The flow rate was 1.0 mL/min. The sample injection volume was 60 µL. The DAD detector monitored peaks by adsorption at 210 nm for thromboxane. The quantitation was based on peak areas with internal standard calibration. ChemStation Software was used for the quantitative analysis (Agilent Technologies, Cheadle, UK) [24].

### 2.3. Statistical Analysis

Statistica 12.0 (Statsoft, Kraków, Poland) was used to perform the calculation dates. As the distribution in most cases deviated from normal, non-parametric tests were used: Mann–Whitney. For comparisons between groups (DIP vs. NDIP and gender), *p* < 0.05 was considered statistically significant. A significance level of *p* = 0.051–0.099 was designated as a trend at the limit of statistical significance.

## 3. Results

The group of patients with ischemic stroke (*n* = 62) was differentiated in terms of gender (Table 1). Only in the case of hemoglobin (HB) and the number of erythrocytes (Ery) did they differ statistically significantly (*p* = 0.039 *p* = 0.051). The trend, i.e., *p* < 0.1, was observed with respect to BMI, HCT and fasting glucose (Table 1).

Analyzing the group of patients after ischemic stroke, taking into account the presence of the phenomenon of dipping or its absence, it was found that the groups differed in terms of glucose levels in fasting (Table 2). Nondipping patients had a statistically significantly lower glucose level averaging 114.94 ± 17.48. The other parameters did not differ statistically significantly.

After taking into account gender in the DIP and NDIP groups, it was found that there were no significant differences in the group of women in relation to the parameters tested (Table 3). However, in the group of men, there were significant differences in relation to diastolic pressure, which was higher in the DIP group, averaging 152.67 ± 16.50 mmHg, than in the NDIP group (134.73 ± 25.75 mmHg). Additionally, in the NDIP group, a higher number of platelets was observed at the borderline of significance. However, the platelet count was comparable to both groups of women (Table 3).

The analysis of the thromboxane level in the plasma of ischemic stroke patients showed significant differences in terms of sex (Table 4A). The mean percentage decrease in blood pressure during the night period for both systolic (DIP Sys%) and diastolic (DIP Dia%) blood pressure was not significant with respect to sex. After dividing the patients into people with the existing DIP and NDIP phenomena, it was found that the thromboxane level did not differ between the groups, but Dip SyS% and Dip Dia% differed significantly (Table 4B). The last step was the division of the DIP and NDIP groups by gender (Table 4C). There was a trend in the male group and a lower thromboxane level in the male DIP group. The DIP Sys% and DIP Dia% values in both groups (DIP, NDIP) also differed significantly in terms of gender (Table 4C).

## 4. Discussion

It is believed that thromboembolic disorders underlie the pathogenesis of ischemic stroke [25]. The use of thromboxane A2 receptor antagonists has a positive effect in the treatment of various types of cerebrovascular diseases [26]. Due to this mechanism, we were interested in whether the DIP phenomenon is related to the arachidonic acid cascade. One of the phospholipases (Phospholipase C) causes the opening of calcium channels and leads to their release from intraplate granules under the influence of factors intensifying oxidative stress. The increase in the concentration of calcium ions activates phospholipase A2, which hydrolyzes ester bonds between unsaturated fatty acids and glycerol [27].

The AA released in this way is converted via the constitutive COX1 pathway into metabolites such as thromboxane A2 or prostaglandin H2, which mainly activate platelets. This leads to a change in their shape and the aggregation and narrowing of blood vessels. Thus, once started, the hydrolysis reaction can act as a self-directed and self-propelling process leading to an increasingly stronger activation of platelets, their grouping and, consequently, the formation of a blood clot [26].

Thromboxane B2 is strongly involved in the atherosclerotic process, because in addition to activating platelets, it also has a prescription in cells of the immune system (monocytes, macrophages and lymphocytes), as well as in smooth muscle cells and endothelial cells. It has been shown that the inhibition of the TXA receptor (TPRs) not only reduces plaque formation but can also induce its regression [28]. Pharmacological lowering of TXA2 level causes, inter alia, lowering blood pressure. However, the relationship between the TXA2 level and the Non-DIP phenomenon has not been clarified, although our research indicates a relationship between these parameters. Interestingly, in our study, the differences in TXA levels were not statistically significant between the DIP vs. Non-DIP. Only the division into sex revealed differences in its level, as well as in the amount of PLT and diastolic pressure. We observed lower blood levels of both TXA and PLT, with a higher diastolic blood pressure index, in women with Non-DIP vs. DIP women, but in men, we observed exactly the opposite relationship: higher levels of TXA and PLN, with lower diastolic pressure. Available scientific research recognizes that male gender is a potent risk factor for stroke. Appelros et al. (2009) indicate a 33% higher risk of stroke in men than in women. On the other hand, a stroke in women is usually more severe and has an increased mortality (24.7% in one month) compared to men (19.7%). Therefore, gender seems to be of much greater importance in the clinical course of stroke and prognosis, but there are no clear clinical guidelines for different patient management depending on gender [29,30]. The gender effect of ischemic stroke and the nondipping phenomena that predispose to stroke are not yet well understood. This is due to the complexity of both processes and their multifactorial nature [31,32].

The modifiable factor that differentiated the Non-DIP men and women from the control groups was BMI. Here, as in the case of TXA, the differences were only visible after the division into sex. Non-DIP women were characterized by a higher BMI than the control, and non-DIP men lower. BMI is a measure of weight to height; it does not distinguish between fat and lean mass [33], but we suspect that these differences may be due to the content of body fat, which is related to gender. However, this is only a guess, because this parameter has not been tested. In general, men have higher muscle mass than women, so in women, the intensity of inflammation modulated by adipose tissue may be higher.

Large cohort studies conducted in the UK show that increased BMI in women is associated with an increased risk of ischemic stroke (relative risk 1.21 per 5 kg/m^2^ BMI, 95% confidence interval 1.18–1.23, *p* < 0.0001) but reduced risk of hemorrhagic stroke (relative risk 0.89 per 5 kg m^2^ BMI, 0.86–0.92, *p* < 0.0001). The reasons for the difference between the two types of stroke related to BMI are unclear, although the authors see it in an increased apolipoprotein B/A1 ratio in people with abnormal BMI. BMI increases the risk of ischemic stroke through altered lipid levels [34] and is a risk factor for the Non DiP phenomenon [35]. Our observations of BMI in women Dip vs. Non-DIP conforms to the above.

Obesity is associated with well-known risk factors for stroke, including hypertension, dyslipidemia and diabetes, but is also considered to be one of the main causes of the nondipping phenomenon (along with endocrine disorders, kidney dysfunction and autonomic dysfunction) [36]. Nonsubmerging pressure is more common in people with metabolic syndrome. Higher diurnal blood pressure may increase markers of overall cardiovascular stress already associated with metabolic syndrome [37].

There are several known hypotheses linking hypertension and obesity, such as the activation of the RAAS system, hypoglycemia, the activation of the immune system, oxidative stress and low-grade inflammation [38,39]. There is also a similarity in the mechanisms of obesity-induced hypertension and non-submerging causes [17]. Inflammation and oxidative stress in adipose tissue play a very important role in the interactions of microvascular endothelium in obesity-related hypertension. Adipose tissue produces a range of compounds such as leptin, resistin, adiponectin, and visphatin, as well as cytokines such as TNF-α, IL-6, MCP-1, and IL-1 that regulate adipose tissue inflammation [38]. IL6 in particular is considered a prognostic marker in ischemic stroke. It can also be used both to monitor stroke recurrence as well as be a predictor for patients at high risk of stroke [40,41]. To the best of our knowledge, there are no literature data describing the level of IL6 in the non-dip phenomenon. However, significantly higher levels of IL6 are observed in people who develop cardiovascular disease (CVD) [42]. It has also been shown that interleukin-6 biosynthesis can be promoted by thromboxane A2 in glial cells [43]. Microglia is the main source of brain TXA2, binding to a specific G protein-coupled receptor. The distribution of TXA2 receptors in the brain indicates that it may be involved in many diseases of the central nervous system [44]. In the brain, the second COX2 pathway is activated, which is responsible for the proper functioning of the CNS and for such brain functions as synaptic activity. This pathway also produces thromboxane A2 (TXA2) together with prostacyclin (PGI2). Under physiological conditions, PGI2 and TXA2 are highly expressed in the cerebral cortex and the hippocampus. Both metabolites are also involved in the pathophysiological processes of ischemic brain damage [45]. A balance between prostacyclin (PGI2) and TXA2 is important to protect neurons. The use of COX2 inhibitors that selectively inhibit PGI2 without concurrently TAX2 inhibition may be responsible for hypertension, thrombosis and increasing cardiovascular risk [46]. Understanding the molecular cascade leading to stroke is still ahead of us. However, our observations on the level of TXA in the non-dipping phenomenon significantly enrich the knowledge on this topic.

Physiologically, an ischemic stroke is preceded by damage to the endothelium of the brain′s blood vessels, which causes inflammation and the deposition of fatty deposits in the artery walls. A subsequent reduction in blood vessel diameter and vascular flow causes states of hypoxia and hypoglycemia in the brain tissue [47] due to the fact that, in the authors′ research, it was found that higher fasting glucose levels were recorded among DIP men (trend *p* = 0.093; Table 2). It can be assumed that disturbances in carbohydrate metabolism leading to an increase in blood glucose levels with simultaneous hypoglycemia in peripheral tissues, including brain tissue, are an important stage leading to a stroke event. Contrastingly, in the NDIP group, carbohydrate disturbances may be a less important factor. Moreover, the NDIP group included men 10 years younger on average with lower BMI and lower diastolic pressure but higher PLT.

Therefore, we are inclined to say that in this group, inflammation involving TX and NDIP were a more important factor in the occurrence of stroke than disturbances in carbohydrate metabolism. High TX levels may be related to the harmful phenomenon of non-dipping in both men and women. In our study of women, DIP vs. DIP men and Non-DIP women vs. Non-DIP men were characterized by a higher TXA level.

Probably TXA2 itself is not the main determinant of the DIP phenomenon, but possibly one of the more important. Of course, other pro-inflammatory factors may also be involved in the occurrence of this singularity, supporting the thesis of other researchers on the role of gender in the pathogenesis of stroke, including the presence of inflammation [48]. We believe that gender should be more carefully considered, both in stroke prevention and in stroke itself.

The limitation of the study is a relatively small group of patients from one center (ethnically homogeneous Caucasian race). We suppose that the potential impact of the measurement time from the moment of stroke appears to be small.

## 5. Conclusions

Despite many years of research into the non-immersion pattern, there is no single clear mechanism for its pathology. High TX levels may be related to the harmful phenomenon of nondipping in both men and women. Especially in women in both DIP/NDIP groups, the concentration of TXA2 was higher than in the group of men, although NDIP men had higher TXA2 levels than DIP men. Further research on the role of gender in the pathogenesis of stroke and in the presence of inflammation seems advisable. TXA2 is an important metabolite of AA, but other pro-inflammatory factors may also be involved in the occurrence of this singularity.

## Figures and Tables

**Table 1:** Characteristics of the study group including gender.

Parameter	Stroke Total Avg ± SD *n* = 62	Women Avg ± SD *n* = 34	Men Avg ± SD *n* = 28	*p*-Value
Age (years)	60.64 ± 12.10	62.29 ± 11.08	58.64 ± 13.17	0.240
BMI (kg/m^2^)	28.48 ± 4.77	27.49 ± 4.46	29.67 ± 4.94	0.073
CRP (mg/L)	2.60 ± 3.98	2.38 ± 2.18	1.92 ± 1.68	0.363
Total cholesterol (mg/dL)	199.93 ± 53.29	213.71 ± 58.56	183.14 ± 41.16	0.220
LDL-C (mg/dL)	116.76 ± 44.86	123.85 ± 58.56	108.14 ± 37.75	0.444
HDL (mg/dL)	51.90 ± 15.22	55.50 ± 15.66	47.54 ± 13.70	0.270
TG (mg/dL)	158.69 ± 78.5	174.35 ± 89.87	139.68 ± 58.02	0.282
Non-HDL (mg/dL)	146.47 ± 51.90	154.25 ± 59.32	137.01 ± 40.04	0.534
Systolic pressure (mmHg)	145.03 ± 20.33	144.56 ± 19.12	145.61 ± 22.05	0.842
Diastolic pressure (mmHg)	87.98 ± 14.44	88.23 ± 11.21	87.68 ± 17.82	0.881
HB (g/dL)	14.44 ± 1.13	13.92 ± 0.92	15.07 ± 1.02	0.039
HCT (%)	41.55 ± 3.09	40.43 ± 2.95	42.92 ± 2.74	0.085
ERY (10^12^/L)	4.74 ± 0.40	4.58 ± 0.37	4.94 ± 0.34	0.050
WBC (10^9^/L)	8.55 ± 2.10	8.95 ± 1.98	8.06 ± 2.18	0.444
PLT (10^9^/L)	238.73 ± 81.72	256.03 ± 97.65	217.71 ± 50.98	0.276
Glucose (mg/dl)	125.81 ± 41.37	117.79 ± 30.25	135.54 ± 50.68	0.093
Diabetes (*n*)	30	16	14	-
Ischaemic heart disease (*n*)	6	5	1	-
Hypertension (*n*)/hypotensive drugs (*n*)	50/34	28/22	22/12	-
Smoking	23	14	9	-

BMI—Body Mass Index; CRP—*C*-reactive protein; LDL-C—low-density lipoprotein; HDL—high-density lipoprotein; TG—triglycerides; Non-HDL—non-high-density lipoprotein; HB—hemoglobin; HCT—hematocrit; EBC—erythrocytes; WBC—white blood cells; PLT—platelets.

**Table 2:** Characteristics of the DIP and NDIP groups excluding gender.

Parameter	DIP Avg ± SD *n* = 31	Non-DIP Avg ± SD *n* = 33	*p*-Value
Gender *n* (Women and Men)	W = 14 M = 17	W = 22 M = 11	-
Age (years)	62.39 ± 11.95	58.45 ± 12.35	0.201
BMI (kg/m^2^)	29.13 ± 4.17	27.88 ± 5.19	0.291
CRP (mg/L)	2.14 ± 2.08	2.91 ± 5.11	2.083
Total cholesterol (mg/dL)	195.94 ± 57.36	204.48 ± 48.03	0.441
LDL-C (mg/dL)	112.45 ± 47.62	120.70 ± 41.23	0.519
HDL (mg/dL)	50.97 ± 14.11	54.36 ± 17.45	0.461
TG (mg/dL)	162.58 ± 83.58	151.82 ± 72.90	0.397
Non-HDL (mg/dL)	139.25 ± 52.16	153.76 ± 49.77	0.584
Diastolic pressure (mm Hg)	148.39 ± 17.91	143.09 ± 23.16	0.312
Systolic pressure (mmHg)	87.90 ± 11.31	88.79 ± 16.91	0.808
HB (g/dL)	14.49 ± 1.24	14.309 ± 1.043	0.521
HCT (%)	41.90 ± 3.44	41.006 ± 2.757	0.254
ERY (10^12^/L)	4.73 ± 0.42	4.721 ± 0.393	0.936
WBC (10^9^/L)	8.35 ± 1.86	8.696 ± 2.301	0.514
PLT (10^9^/L)	227.29 ± 101.26	253.64 ± 55.337	0.197
Glucose (mg/dL)	137.03 ± 54.07	114.97 ± 17.438	0.030
Diabetes (*n*)	15	15	-
Ischemic heart disease (*n*)	3	3	-
Hypertension (*n*)/hypotensive drugs (*n*)	27/18	23/16	-
Smoking	9	14	-

BMI—Body Mass Index; CRP-*C*—reactive protein; LDL-C—low-density lipoprotein; HDL—high-density lipoprotein; TG—triglycerides; Non-HDL—non-high-density lipoprotein; HB—hemoglobin; HCT—hematocrit; EBC—erythrocytes; WBC—white blood cells; PLT—platelets.

**Table 3:** Characteristics of the DIP and NDIP groups including gender.

Parameter	Women DIP Avg ± SD *n* = 14	Women Non-DIP Avg ± SD *n* = 20	Men DIP Avg ± SD *n* = 17	Men Non-DIP Avg ± SD *n* = 11	*p*-Value *	*p*-Value **
Thromboxane (µg/mL)	0.0109 ± 0.38	0.0094 ± 0.007	0.0053 ± 0.0029	0.0068 ± 0.0038	0.513	0.090
Age (years)	62.29 ± 11.95	62.30 ± 10.74	62.47 ± 12.31	52.73 ± 12.75	0.997	0.054
BMI (kg/m^2^)	26.77 ± 4.45	27.99 ± 4.51	31.06 ± 5.056	27.53 ± 4.08	0.439	0.063
CRP (mg/L)	2.23 ± 2.52	2.49 ± 1.94	2.07 ± 1.722	1.69 ± 1.68	0.734	0.569
Total cholesterol (mg/L)	204.14 ± 72.22	220.40 ± 47.66	189.17 ± 42.70	173.82 ± 38.74	0.434	0.344
LDL-C (mg/L)	115.43 ± 58.95	129.75 ± 42.08	110.00 ± 37.61	105.27 ± 39.62	0.414	0.753
HDL (mg/L)	52.500 ± 12.77	57.60 ± 17.41	49.71 ± 15.39	44.18 ± 10.36	0.358	0.306
TG (mg/L)	180.07 ± 105.6	170.35 ± 79.74	148.18 ± 59.59	126.55 ± 55.63	0.761	0.345
Non-HDL	142.69 ± 65.16	164.27 ± 53.99	136.27 ± 39.84	138.37 ± 44.17	0.347	0.908
Diastolic pressure (mm Hg)	143.21 ± 18.77	145.50 ± 19.79	152.67 ± 16.50	134.73 ± 25.75	0.737	0.033
Systolic pressure (mmHg)	88.57 ± 13.07	88.00 ± 10.05	87.35 ± 10.02	88.18 ± 26.39	0.886	0.907
HB (g/dl)	13.88 ± 1.23	13.94 ± 0.74	15.00 ± 1.023	15.17 ± 1.05	0.846	0.670
HCT (%)	40.78 ± 3.88	40.18 ± 2.16	42.82 ± 2.81	43.06 ± 2.74	0.568	0.825
ERY (10^12^/L)	4.54 ± 0.43	4.60 ± 0.34	4.882 ± 0.35	5.026 ± 0.31	0.667	0.277
WBC (10^9^/L)	9.29 ± 1.69	8.71 ± 2.17	7.577 ± 1.67	8.814 ± 2.71	0.408	0.146
PLT (10^9^/L)	256.79 ± 139.7	255.5 ± 56.58	203.00 ± 44.39	240.45 ± 54.09	0.971	0.056
Glukoza (mg/dL)	12,736 ± 43.16	111.10 ± 14.14	145.00 ± 61.80	120.91 ± 20.91	0.125	0.226
Diabetes (*n*)	8	8	10	4	-	-
Ischemic heart disease (*n*)	3	2	1	0	-	-
Hypertension (*n*)/hypotensive drugs (*n*)	11/8	17/14	16/10	6/2	-	-
Smoking	6	9	4	5	-	-

BMI—Body Mass Index; CRP-*C*—reactive protein; LDL-C—low-density lipoprotein; HDL—high-density lipoprotein; TG—triglycerides; Non-HDL—non-high-density lipoprotein; HB—hemoglobin; HCT—hematocrit; EBC—erythrocytes; WBC—white blood cells; PLT—platelets; *—*p*-value women DIP vs. N-DIP; **—*p*-value men DIP vs. N-DIP.

**Table 4:** The level of TX and the value of blood pressure drop during the night ((**A**). including gender; (**B**). including DIP and NDIP; (**C**) taking into account gender in the DIP and NDIP groups).

A.	Stroke Total Avg ± SD *n* = 62	Women Avg ± SD *n* = 34	Men Avg ± SD *n* = 28		*p*-Value	
Thromboxane (µg/mL)	0.0082 ± 0.0059	0.0101± 0.007	0.0059 ± 0.003		0.0004	
DIP Sys%	6.992 ± 7.007	7.532 ± 8.166	6.336 ± 5.347		0.508	
DIP Dia%	9.419 ± 8.170	10.291 ± 9.279	8.361 ± 6.591		0.359	
B.	Grupa DIP avg ± SD *n* = 31		Grupa N- DIP avg ± SD *n* = 33		*p*-value	
Thromboxane (µg/mL)	0.0083 ± 0.058		0.0078 ± 0.059		0.746	
DIP Sys%	1.97 ± 3.93		12.06 ± 5.59		8.45 × 10^−12^	
DIP Dia%	2.87 ± 4.31		15.88 ± 5.25		5.6 × 10^−16^	
C.	Women avg ± SD		Men avg ± SD		*p*-value *	*p*-value **
	DIP *n* = 14	N-DIP *n* = 20	DIP *n* = 17	N- DIP *n* = 11		
Thromboxane (µg/mL)	0.0109 ± 0.38	0.0094 ± 0.007	0.0053 ± 0.003	0.0068 ± 0.004	0.513	0.090
DIP Sys%	−0.19 ± 6.22	12.94 ± 3.82	3.753 ± 4.44	10.33 ± 4.09	1.07 × 10^−8^	0.0005
DIP Dia%	1.42 ± 6.59	16.50 ± 4.61	4.071 ± 3.60	14.99 ± 4.11	5.7 × 10^−9^	7.1 × 10^−8^

*—*p*-value women DIP vs. N-DIP; **—*p*-value men DIP vs. N-DIP.

## Data Availability

The data presented in this study are available on request from the corresponding author. The data are not publicly available due to privacy restrictions.

## References

[1] Fanning J.P., Wong A.A., Fraser J.F. (2014). The epidemiology of silent brainin farction: A systematic review of population-based cohorts. BMC Med..

[2] Easton J.D., Saver J.L., Albers G.W., Alberts M.J., Chaturvedi S., Feldmann E., Hatsukami T.S., Higashida R.T., Johnston S.C., Kidwell C.S. (2009). Definition and evaluation of transient ischemicattack: A scientific statement for health care professionals from the American heart association/American stroke association stroke council; council on cardiovascular surgery and anesthesia; council on cardiovascular radiology and intervention; council on cardiovascular nursing; and the interdisciplinary council on peripheral vascular disease. Stroke.

[3] Cruz-Flores S., Rabinstein A., Biller J., Elkind M.S.V., Griffith P., Gorelick P.B., Howard G., Leira E.C., Morgenstern L.B., Ovbiagele B. (2011). Racial-ethnicdis parities in stroke care: The American experience: A statement for health care professionals from the American Heart Association/American Stroke Association. Stroke.

[4] Agyemang C., Bhopal R., Bruijnzeels M., Redekop W.K.J. (2005). Does nocturnal blood pressure fall in people of African and South Asian descent differ from that in European white populations? A systematic review and meta-analysis. Hypertension.

[5] Gupta A., Baradaran H., Al-Dasuqi K., Knight-Greenfield A., Giambrone A.E., Delgado D., Wright D., Teng Z., Min J.K., Navi B.B. (2016). Gadolinium Enhancement in Intracranial Atherosclerotic Plaque and Ischemic Stroke: A Systematic Review and Meta-Analysis. J. Am. Heart Assoc..

[6] Bonaventura A., Liberale L., Vecchié A., Casula M., Carbone F., Dallegri F., Montecucco F. (2016). Update on inflammatory biomarkers and treatments in ischemic stroke. Int. J. Mol. Sci..

[7] Sacco R.L., Kasner S.E., Broderick J.P., Caplan L.R., Connors J.J.B., Culebras A., Elkind M.S.V., George M.G., Hamdan A.D., Higashida R.T. (2013). An updated definition of stroke for the 21st century: A statement for health care professionals from the American heartassociation/American strokeassociation. Stroke.

[8] Russo I., Viretto M., Barale C., Mattiello L., Doronzo G., Pagliarino A., Cavalot F., Trovati M., Anfossi G. (2012). High glucose inhibits the aspirin-induced activation of the nitricoxide/cGMP/cGMP-dependent protein kinase pathway and does not affect the aspirin-induced inhibition of thromboxane synthesis in humanplatelets. Diabetes.

[9] Lee D.W., Gardner R., Porter D.L., Louis C.U., Ahmed N., Jensen M., Grupp S.A., Mackall C.L. (2014). Currentconcepts in the diagnosis and management of cytokinereleasesyndrome. Blood.

[10] Vandvik P.O., Lincoff A.M., Gore J.M., Gutterman D.D., Sonnenberg F.A., Alonso-Coello P., Akl E.A., Lansberg M.G., Guyatt G.H., Spence F.A. (2012). Primary and secondary prevention of cardiovascular disease: Antithrombotic therapy and prevention of thrombosis, 9th ed: American College of chest physiciansevidence-based clinical practice guidelines. Chest.

[11] Smith S.M., Soubhi H., Fortin M., Hudon C., O’Dowd T. (2012). Managing patients with multimorbidity: Systematic review of interventions in primary care and community settings. BMJ.

[12] Szczuko M., Kozioł I., Kotlega D., Brodowski J., Drozd A. (2021). The Role of Thromboxane in the Course and Treatment of Ischemic Stroke: Review. Int. J. Mol. Sci..

[13] Iannucci G., Petramala L., La Torre G., Barbaro B., Balsano C., Curatulo P.G., Amadei F., Paroli M., Concistrè A., Letizia C. (2017). Evaluation of tolerance to ambulatory blood pressure monitoring: Analysis of dipping profile in a large cohort of hypertensivepatients. Medicine.

[14] Kario K., Kanegae H., Tomitani N., Okawara Y., Fujiwara T., Yano Y., Hoshide S. (2019). Nighttime Blood Pressure Measured by Home Blood Pressure Monitoring as an Independent Predictor of Cardiovascular Events in General Practice. Hypertension.

[15] Mann S., Altman D.G., Raftery E.B., Bannister R. (1983). Circadian variation of blood pressure in autonomic failure. Circulation.

[16] Biaggioni I. (2008). Circadian Clocks, Autonomic Rhythms And Blood Pressure Dipping. Hypertension.

[17] Dubielski Z., Zamojski M., Wiechecki B., Możeńska O., Petelczyc M., Kosior D.A. (2016). The current state of knowledge about the dipping and non-dipping hypertension. Art. Hypertens..

[18] Hoshide S., Kario K., Hoshide Y., Umeda Y., Hashimoto T., Kunii O., Ojima T., Shimada K. (2003). Associations between nondipping of nocturnal blood pressure decrease and cardiovascular target organ damage in strictly selected community-dwelling normotensives. Am. J. Hypertens..

[19] Cuspidi C., Sala C., Tadic M., Gherbesi E., Grassi G., Mancia G. (2016). Nondipping pattern and carotid atherosclerosis: A systematic review and meta-analysis. J. Hypertens..

[20] Cuspidi C., Tadic M., Sala C., Carugo S., Mancia G., Grassi G. (2021). Reverse dipping and subclinical cardiac organ damage: A meta-analysis of echocardiographic studies. J. Hypertens..

[21] Cuspidi C., Tadic M., Sala C., Gherbesi E., Grassi G., Mancia G. (2019). Blood Pressure Non-Dipping and Obstructive Sleep Apnea Syndrome: A Meta-Analysis. J. Clin. Med..

[22] Adams H.P., del Zoppo G., Alberts M.J., Bhatt D.L., Brass L., Furlan A., Grubb R.L., Higashida R.T., Jauch E.C., Kidwell C. (2007). The American Academy of Neurology affirms the value of this guideline as an educational tool for neurologists. Circulation.

[23] Turak O., Ozcan F., Tok D., Işleyen A., Sökmen E., Taşoğlu I., Aydoğdu S., Sen N., McFann K., Johnson R.J. (2013). Serum uric acid, inflammation, and nondipping circadian pattern in Essentials hypertension. J. Clin. Hypertens..

[24] Szczuko M., Kotlęga D., Palma J., Zembroń-Łacny A., Tylutka A., Gołąb-Janowska M., Drozd A. (2020). Lipoxins, RevD1 and 9, 13 HODE as the most import ant derivatives after an Elary incident of ischemic stroke. Sci. Rep..

[25] Bala M.M., Celinska-Lowenhoff M., Szot W., Padjas A., Kaczmarczyk M., Swierz M.J., Undas A. (2020). Antiplatelet and anticoagulant agents for secondary prevention of stroke and other thromboembolic events in people with antiphospholipid syndrome. Cochrane Database. Syst. Rev..

[26] Bots M.L., Ford I., Lloyd S.M., Laurent S., Touboul J.P., Hennerici M.G. (2014). Thromboxane prostaglandin receptor antagonist and carotid atherosclerosis progression in patients with cerebrovascular disease of ischemic origin: A randomized controlled trial. Stroke.

[27] Schaeffer E.L., Forlenza O.V., Gattaz W.F. (2009). Phospholipase A2 activation as a therapeutic approach for cognitive enhancement in early-stage Alzheimer disease. Psychopharmacology.

[28] Davì G., Santilli F., Vazzana N. (2012). Thromboxane receptors antagonists and/or synthase inhibitors. Handb. Exp. Pharmacol..

[29] Appelros P., Stegmayr B., Terént A. (2009). Sex differences in stroke epidemiology: A systematic review. Stroke.

[30] Cao M., Li B., Rong J., Li Q., Sun C. (2022). Sex differences in global disability-adjusted life years due to ischemic stroke: Findings from global burden of diseases study 2019. Sci. Rep..

[31] Reeves M.J., Bushnell C.D., Howard G., Gargano J.W., Duncan P.W., Lynch G., Khatiwoda A., Lisabeth L. (2008). Sex differences in stroke: Epidemiology, clinical presentation, medical care, and outcomes. Lancet. Neurol..

[32] Phan H.T., Gall S.L., Blizzard C.L., Lannin N.A., Thrift A.G., Anderson C.S., Kim J., Grimley R.S., Castley H.C., Kilkenny M.F. (2021). AuSCR Consortium, Stroke123 Investigators. Sex differences in quality of life after stroke were explained by patient factors, not clinical care: Evidence from the Australian Stroke Clinical Registry. Eur. J. Neurol..

[33] Stein C.J., Colditz G.A. (2004). The epidemic of obesity. J. Clin. Endocrinol. Metab..

[34] Kroll M.E., Green J., Beral V., Sudlow C.L., Brown A., Kirichek O., Price A., Yang T.O., Reeves G.K. (2016). Million Women Study Collaborators. Adiposity and ischemic and hemorrhagic stroke: Prospective study in women and meta-analysis. Neurology.

[35] Gavriilaki M., Anyfanti P., Nikolaidou B., Lazaridis A., Gavriilaki E., Douma S., Gkaliagkousi E. (2020). Nighttime dipping status and risk of cardiovascular events in patients with untreated hypertension: A systematic review and meta-analysis. J. Clin. Hypertens..

[36] Moczulska B., Zechowicz M., Leśniewska S., Osowiecka K., Gromadziński L. (2020). The Impact of Obesity on Nighttime Blood Pressure Dipping. Medicina.

[37] Hassan M.O., Jaju D., Albarwani S., Al-Yahyaee S., Al-Hadabi S., Lopez-Alvarenga J.C., Rizvi S.G., Comuzzie A.G., Bayoumi R.A. (2007). Non-dipping blood pressure in the metabolic syndrome among Arabs of the Oman family study. Obesity.

[38] Kang Y.S. (2013). Obesity associated hypertension: New insights into mechanism. Electrolyte Blood Press..

[39] Petrie J.R., Guzik T.J., Touyz R.M. (2018). Diabetes, Hypertension, and Cardiovascular Disease: Clinical Insights and Vascular Mechanisms. Can. J. Cardiol..

[40] Aref H.M.A., Fahmy N.A., Khalil S.H., Ahmed M.F., ElSadek A., Abdulghani M.O. (2020). Role of interleukin-6 in ischemic stroke outcome. Egypt J. Neurol. Psych. Neurosurg..

[41] Zhu H., Hu S., Li Y., Sun Y., Xiong X., Hu X., Chen J., Qiu S. (2022). Interleukins and Ischemic Stroke. Front. Immunol..

[42] Zhang B., Li X.L., Zhao C.R., Pan C.L., Zhang Z. (2018). Interleukin-6 as a Predictor of the Risk of Cardiovascular Disease: A Meta-Analysis of Prospective Epidemiological Studies. Immunol. Investig..

[43] Obara Y., Kurose H., Nakahata N. (2005). Thromboxane A2 promotes interleukin-6 biosynthesis mediated by an activation of cyclic AMP-response element-binding protein in 1321N1 human astrocytoma cells. Mol. Pharmacol..

[44] Yang W., Yan A., Zhang T., Shao J., Liu T., Yang X., Xia W., Fu Y. (2016). Thromboxane A2 Receptor Stimulation Enhances Microglial Interleukin-1β and NO Biosynthesis Mediated by the Activation of ERK Pathway. Front. Aging Neurosci..

[45] Yu L., Yang B., Wang J., Zhao L., Luo W., Jiang Q., Yang J. (2014). Time course change of COX2-PGI2/TXA2 following global cerebral ischemia reperfusion injury in rat hippocampus. Behav. Brain Funct..

[46] Cannon C.P., Cannon P.J. (2012). Physiology. COX-2 inhibitors and cardiovascular risk. Science.

[47] Hermanides J., Plummer M.P., Finnis M., Deane A.M., Coles J.P., Menon D.K. (2018). Glycaemic control targets after traumatic brain injury: A systematic review and meta-analysis. Crit. Care..

[48] Spychala M.S., Honarpisheh P., McCullough L.D. (2017). Sex Differences in Neuroinflammation and Neuroprotection After Ischemic Stroke. J. Neurosci. Res..

